# A case of coexistence of spinal intradural capillary hemangioma and craniocervical junction dural arteriovenous fistula

**DOI:** 10.1007/s13760-026-03126-5

**Published:** 2026-07-02

**Authors:** Satoshi Kawajiri, Makoto Isozaki, Ken Matsuda, Hidetaka Arishima, Kenichiro Kikuta

**Affiliations:** Department of Neurosurgery, Division of Medicine, Faculty of Medical Sciences, University of Fukui, 23-3 Matsuoka Shimoaizuki, Eiheiji, 910-1193 Fukui Japan

**Keywords:** Capillary hemangioma, Dural arteriovenous fistula, Craniocervical junction, Spinal cord tumor

## Introduction

Capillary hemangiomas (CHs) are benign vascular neoplasms that predominantly involve the cutaneous and soft tissue compartments of the head and neck, particularly in the pediatric population. Although their occurrence in the central nervous system has been increasingly documented, it remains a rare clinical entity. While other vascular malformations such as cavernous malformations are frequently associated with developmental venous anomalies, such a co-occurrence has not been previously documented in spinal CHs. Here, we present the first case of cervical intradural extramedullary CH occurring concurrently with a craniocervical junction dural arteriovenous fistula (CCJ-DAVF) and discuss the potential pathogenetic links.

## Case presentation

**Fig. 1:**
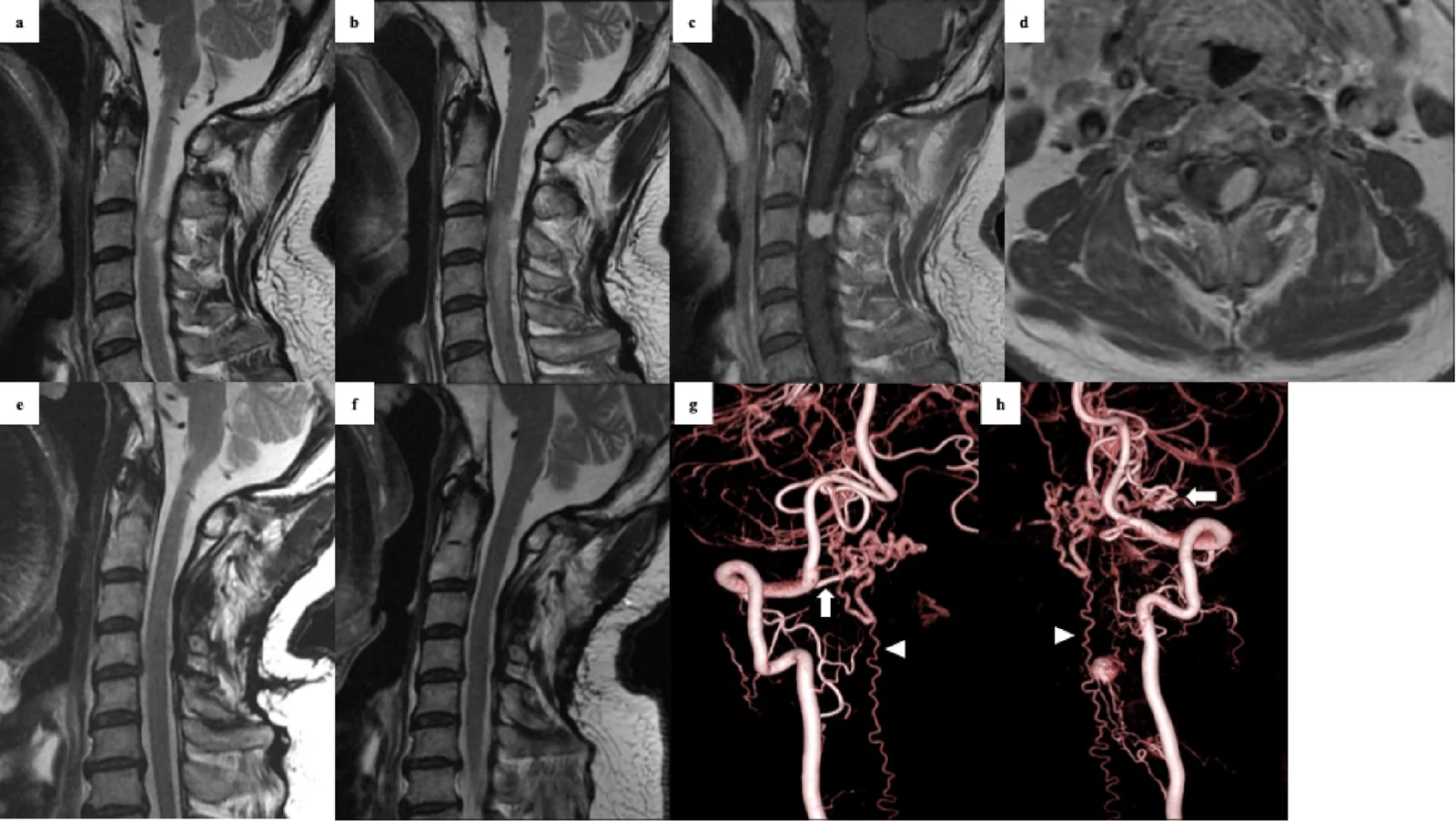
T2-weighted sagittal image showing a hyperintense intradural extramedullary mass at the C3 level with increased surrounding signal (**a, b**) and flow voids along the CCJ (**b**). T1-weighted images with contrast medium show a homogeneously enhanced mass on the left side of the spinal cord (**c, d**). T2-weighted imaging performed 2 years after surgery revealed no residual tumor (**e**), resolution of the hyperintense signal around the tumor, and reduction in flow voids at the CCJ (**f**). Right vertebral angiography (**g**) and left vertebral angiography (**h**) show a CCJ-DAVF supplied by the dural branches of the bilateral vertebral arteries (arrow) draining into the posterior spinal vein (arrow head). The tumor is supplied by the left C5/6 radicular artery (**h**)

A 60-year-old man with no significant medical history presented with a two-month history of progressive posterior neck pain and right numbness. Neurological examination revealed hyperreflexia in the lower extremities and moderate deep sensory impairment (Modified McCormick Scale [mMS] Grade III), although gross motor strength was preserved. Magnetic resonance imaging (MRI) of the cervical spine revealed a well-defined intradural extramedullary lesion at the C3 level, positioned on the left side of the spinal cord. The lesion demonstrated hyperintensity on T2-weighted imaging and homogeneous enhancement after the administration of gadolinium contrast medium. Notably, the tumor was accompanied by intramedullary T2 hyperintensity extending from C3 to C5, indicative of congestive myelopathy or peritumoral edema. Furthermore, prominent flow voids were visualized along the dorsal aspect of the spinal cord and CCJ (Fig. 1). Digital subtraction angiography was subsequently performed to further characterize the vascular architecture. We identified a CCJ-DAVF supplied by the dural branches of the bilateral vertebral arteries, draining inferiorly into the posterior spinal vein. The tumor was hemodynamically distinct from the CCJ-DAVF, supplied by the left C5/6 radicular artery, and drained into the local epidural venous plexus (Fig. 1). Preoperative imaging suggested a spinal tumor, such as a meningioma, schwannoma, or capillary hemangioma, coexisting with the CCJ-DAVF. CCJ-DAVF was considered incidental because no associated signal intensity changes were observed in the medulla or cervical cord.

**Fig. 2:**
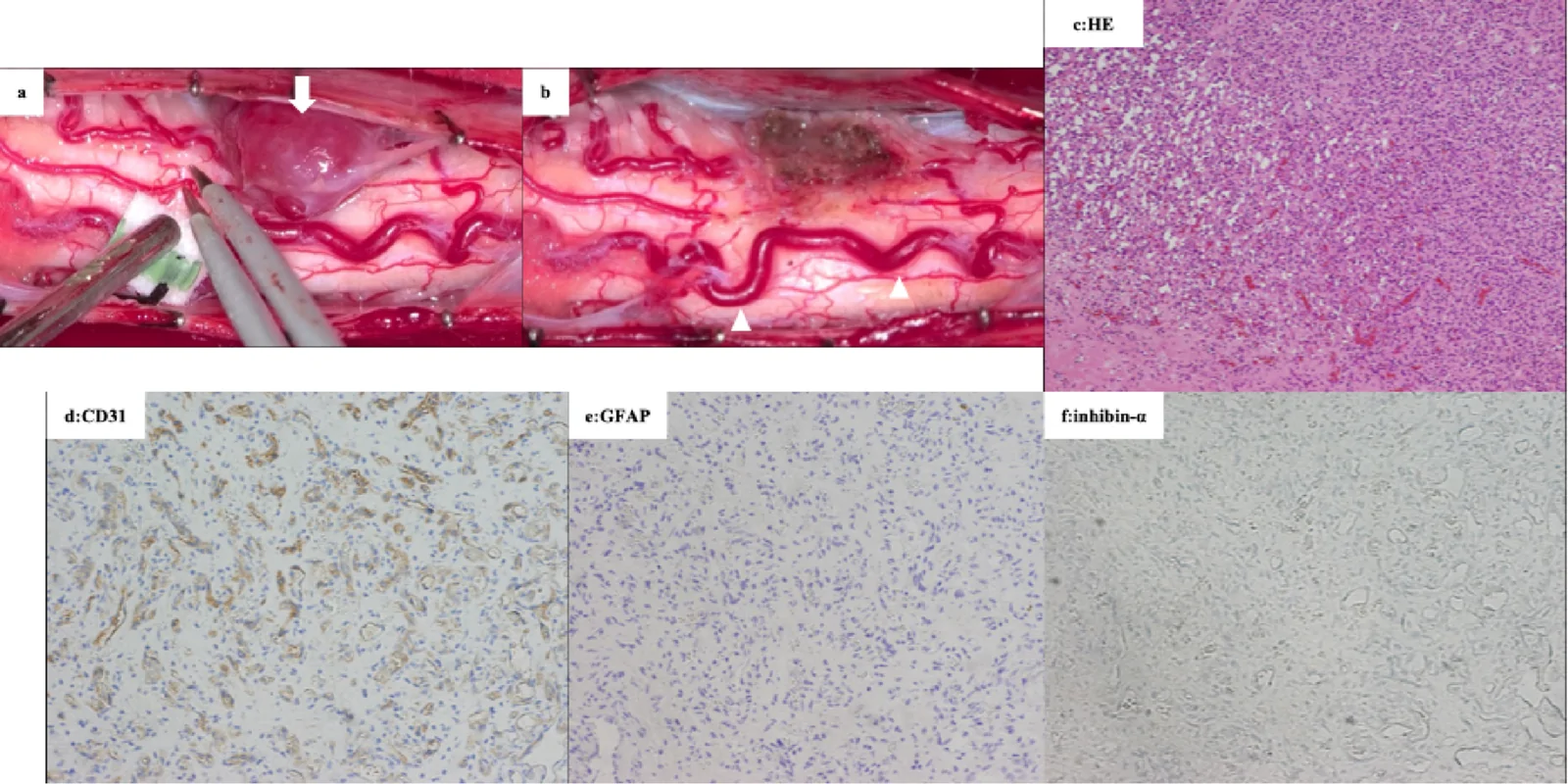
After dural incision, the reddish tumor (arrow) and feeding arteries were identified (**a**). After the tumor dissection, the posterior spinal vein was preserved (arrow head) (**b**). Histological examination revealed a dense proliferation of capillary-sized vessels lined by a single layer of endothelial cells (**c**) at 100* magnification. Immunohistochemical staining was positive for CD31 (**d**) but negative for GFAP (**e**) and inhibin-α (**f**) at 200* magnification

Owing to the neurological deficits primarily caused by direct spinal cord compression by the tumor, surgical resection was prioritized. Following C2–C4 laminectomy and dural incision, a well-circumscribed reddish hypervascular lesion was identified in the left dorsolateral aspect of the spinal cord. A dilated posterior spinal vein was also observed. Intraoperative indocyanine green fluorescence videoangiography confirmed robust internal tumor vascularity. After devascularizing the feeders originating from the right C5/6 radicular artery, the tumor was resected en bloc. The posterior spinal vein was meticulously preserved to avoid exacerbating venous congestion (Fig. 2). Histological examination revealed a dense proliferation of capillary-sized vessels lined by a single layer of endothelial cells. Immunohistochemical staining was positive for CD31 and negative for glial fibrillary acidic protein (GFAP) and inhibin-α, confirming the diagnosis of capillary hemangioma (Fig. 2).

Postoperative MRI showed resolution of the intramedullary T2 hyperintensity and peritumoral flow voids at the C3 level. Intriguingly, a reduction in the flow voids at the CCJ was also observed despite the dAVF remaining untreated (Fig. 1). Although numbness in his left lower extremity persisted, the patient’s symptoms improved to mMS Grade II. During the 2.5-year postoperative course, there was no change in his symptoms, and he remained under surveillance for CH recurrence and asymptomatic CCJ-DAVF.

## Discussion

Spinal CHs in adults are uncommon within the neuraxis, and their pathogenesis remains largely speculative as most cases are sporadic [1–3]. The proposed etiologies include trauma, endocrine influences, and inflammatory triggers [4]. While mucocutaneous lobular CHs have been linked to neoplastic processes involving RAS or BRAF mutations, spinal CHs are hypothesized to arise from dysembryogenesis—specifically, the aberrant incorporation of mesenchymal tissue into the neural tube during primary neurulation—rather than being true neoplasms [5, 6].

The simultaneous occurrence of spinal CH and CCJ-DAVF are exceedingly rare, suggesting a potential pathogenic link rather than a mere coincidence. We propose two primary hypotheses to explain this association. First, localized venous hypertension induced by spinal CH may have triggered the development of CCJ-DAVFs. Although the two lesions are anatomically separate, increased venous pressure is a recognized factor in the postnatal acquisition of dAVFs. Postoperative MRI revealed resolution of both the peritumoral intramedullary T2 hyperintensity and flow voids in the CCJ. These findings suggested improved venous drainage, supporting the hypothesis of a functional hemodynamic link between the two lesions. Secondly, a shared angiogenic environment, particularly involving vascular endothelial growth factor (VEGF), may be a contributing factor. Evidence suggests that tissue injury triggers the localized secretion of angiogenic factors by inflammatory cells [7]. By accelerating excessive blood vessel growth, these factors are hypothesized to play a key role in the pathogenesis of CH following trauma [4, 8]. Furthermore, in a rat model of venous hypertension, Qiang et al. demonstrated that VEGF overexpression prompted dural angiogenesis, whereas a VEGF receptor antagonist significantly lowered the rate of DAVF induction by suppressing this hypervascularization. Based on these findings, they concluded that the VEGF signaling pathway plays a pivotal role in the pathogenesis of venous hypertension-induced DAVFs, supporting a molecular link between VEGF and DAVF formation [9]. Thus, a localized pro-angiogenic microenvironment could have facilitated the synchronous growth or clinical presentation of both lesions. Although these hemodynamic and molecular hypotheses are theoretically plausible, this report is limited by its single-case nature; thus, a direct causal relationship cannot be definitively established.

## Conclusion

This case represents the first documented coexistence of spinal CH and CCJ-DAVF, thereby expanding the known spectrum of mixed vascular pathologies of the central nervous system. This underscores the necessity for a comprehensive radiological evaluation of the entire neuraxis when a vascular lesion is detected. Recognizing that multiple hemodynamically distinct pathologies can coexist is essential for accurate diagnosis and strategic prioritization of surgical interventions.

## Data Availability

No datasets were generated or analysed during the current study.
